# Comparison of different time intervals between laparoscopic cholecystectomy to endoscopic retrograde cholangiopancreatography for patients with cholecystolithiasis complicated by choledocholithiasis

**DOI:** 10.3389/fsurg.2022.1110242

**Published:** 2023-03-15

**Authors:** Lingbo Hu, Xingpeng Shi, Aidong Wang

**Affiliations:** ^1^ Department of Hepatopancreatobiliary Surgery, Taizhou Hospital of Zhejiang Province Affiliated to Wenzhou Medical University, Zhejiang, China; ^2^ Department of Hepatopancreatobiliary Surgery, Enze Hospital, Taizhou Enze Medical Center (Group), Zhejiang, China

**Keywords:** gallstone, common bile duct stone, endoscopic retrograde cholongiopancreatography, laparoscopic cholecystectomy, timing

## Abstract

**Background:**

Endoscopic retrograde cholangiopancreatography (ERCP) followed by laparoscopic cholecystectomy (LC) is a common strategy for treatment of patients with gallstones with co-existing stones in the common bile duct (CBD). We conducted this study to compare the effect of different time intervals between ERCP and LC.

**Methods:**

A total of 214 patients who underwent elective LC after ERCP for gallstones and CBD stones between January 2015 and May 2021 were retrospectively reviewed. We compared the hospital stay, operation time, perioperative morbidity, and conversion rate to open cholecystectomy, according to the interval between ERCP and ERCP and LC, namely, one day, 2–3 days, and 4 days or more. A generalized linear model was used to analyze the differences among the groups for outcomes.

**Results:**

There were a total of 214 patients with 52, 80, and 82 patients in group 1, group 2, and group 3 respectively. These groups did not differ significantly in terms of major complications or conversion to open surgery (*p* = 0.503 and *p* = 0.358, respectively). The generalized linear model showed that operation times in group 1 and group 2 were similar (odds ratio (OR) 0.144, 95% confidence interval (CI) 12.597, 8.511, *p* = 0.704), while operation time was significantly longer in group 3 than in group 1 (OR 4.005, 95% CI, 0.217, 20.837, *p* = 0.045). Post-cholecystectomy hospital stay was similar among the three groups, while post-ERCP hospital stay was significantly longer in group 3 compared with group 1.

**Conclusion:**

We recommend that LC be performed within three days after ERCP to reduce operating time and hospital stay.

## Introduction

Approximately 4.6%–20.8% of patients with gallstones are detected to have stones in the common bile duct (CBD) during intra-operative cholangiography (1, 2). The European Society of Gastrointestinal Endoscopy (ESGE) guideline recommends that common bile duct stones (CBDSs) may be treated by endoscopic retrograde cholangiopancreatography (ERCP) or surgically with CBD exploration during cholecystectomy (3). Four strategies are widely employed for the management of patients with gallstones and co-existing CBDSs: preoperative ERCP (PreERCP) plus laparoscopic cholecystectomy (LC); LC plus laparoscopic CBD exploration (LCBDE); LC plus intra-operative ERCP (IntraERCP), and LC plus post-cholecystectomy ERCP (PostERCP) (4). Several studies have demonstrated that a one-stage strategy including LCBDE and LC plus intraoperative ERCP is superior to a two-stage strategy (LC plus pre- or postoperative ERCP) in regards to complications, post-cholecystectomy hospitalization, and total costs (5, 6). However, performing LCBDE needs highly skilled and trained operators and is associated with the longest overall operative time as compared with the other procedures. The CBD stone-clearance rate is lowest and the incidence of the biliary leak is highest when adopting LC plus post-cholecystectomy ERCP (4). Further, due to the coordination required for surgeons, endoscopists, operating rooms, and surgical equipment, intra-operative ERCP may be difficult to perform in all hospitals. Thus, pre-operative ERCP followed by LC is a good choice because of its simplicity and accessibility.

However, the suitable interval time from ERCP to LC is still controversial and varies from hours to more than 6 weeks (7–17). It has been reported that delayed LC results in more morbidity and recurrent biliary symptoms (12). On the other hand, early LC can reduce re-admissions with gallstone-related symptoms and is not associated with worse surgical outcomes (8). Hence, early LC after ERCP in the same admission was adopted in our center. This study was conducted to compare the effect of different time intervals from ERCP to LC for patients with gallstones with co-existing CBDSs, in terms of conversion rate to open surgery, post-cholecystectomy complications, and length of hospital stay.

## Patients and methods

We conducted a retrospective cohort study on patients with gallbladder stones with co-existing CBDSs, who underwent ERCP for clearance of CBDSs, followed by laparoscopic cholecystectomy (LC). The study was approved by the Ethics Committee of Enze hospital.

The inclusion criteria were: (1) both gallbladder stones and CBDSs confirmed by imaging (ultrasonography/magnetic resonance cholangiopancreatography/computer tomography alone, or combined); (2) patients who underwent ERCP and LC during the same hospital stay; and (3) patients with acute or chronic cholecystitis or acute cholangitis or mild biliary pancreatitis.

The exclusion criteria were: (1) patients with concomitant intrahepatic duct stones; (2) ERCP failure; (3) patients who underwent LC and other operations simultaneously; (4) patients who underwent LC and ERCP simultaneously.

**Figure 1:**
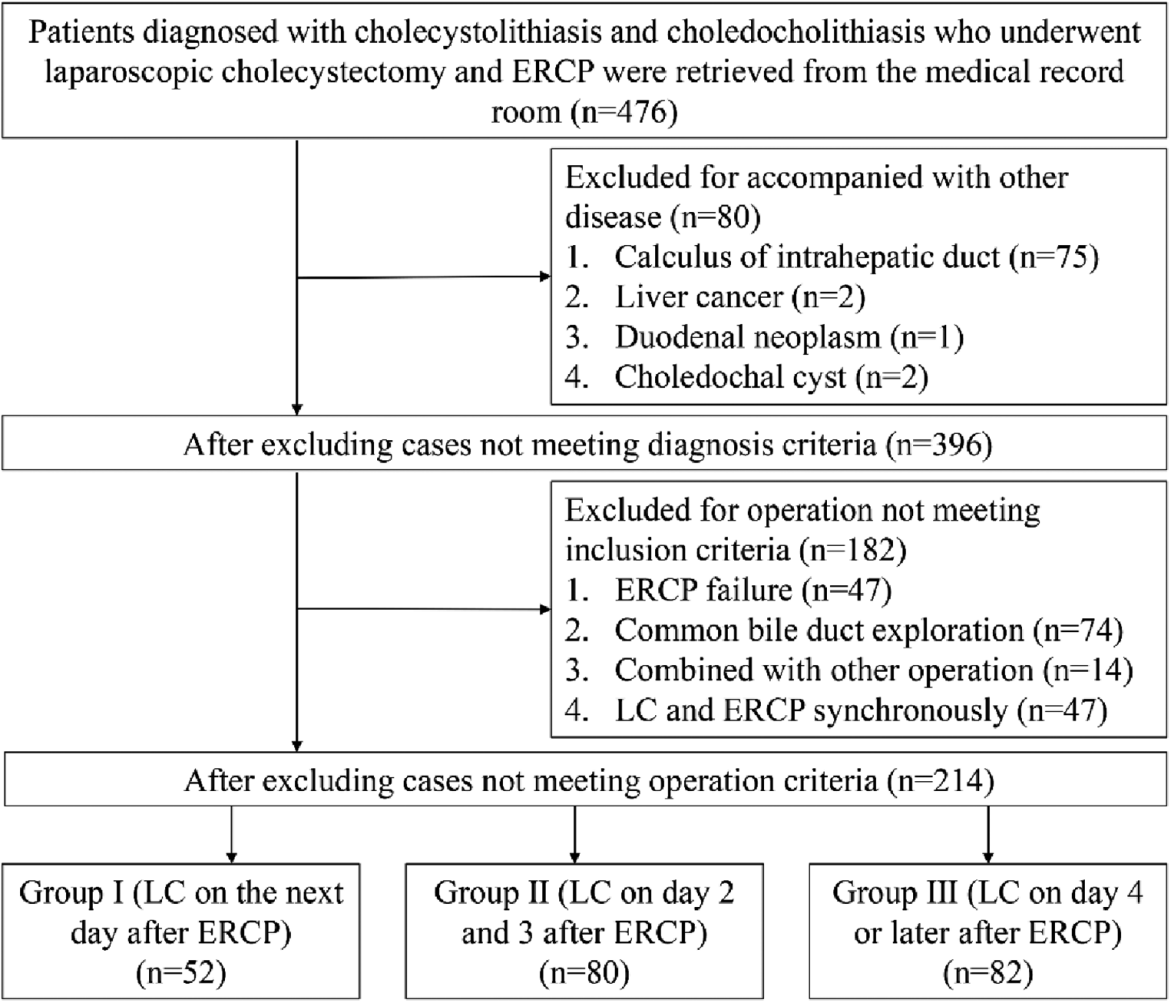
Flow chart of selection process.

Between January 2015 and May 2021, a total of 214 patients at the Enze hospital fulfilled the inclusion criteria and were included in the study. Figure 1. shows the flow chart for selection of patients. Patients were divided into three groups based on the time interval from ERCP to LC. In group 1 (*n* = 52), LC was performed the next day after ERCP. In group 2 (*n* = 80), LC was done on day 2 or day 3 after ERCP, and in group 3 (*n* = 82), LC was performed on day 4 or later after ERCP, but in the same hospitalization. The assignment of patients to different groups depended on logistic issues, such as the availability of facilities (for example, operation was not done on weekends and holidays), and the individual decisions of the patients basing on the tolerance to postoperative discomfort with ERCP.

The following pre-operative parameters were recorded including age, gender, body mass index (BMI), complications, and reports of laboratory examination, and imaging. The three groups were compared statistically in terms of the operation time, post-cholecystectomy length of hospital stay, conversion rate to open surgery, residual calculi, and major morbidities such as biliary duct injury, post-cholecystectomy bleeding, and bile leak.

The ERCP procedure was accomplished with a standard duodenoscope *via* air insufflation (Olympus TJF) under sedation, with selective cannulation of the CBD, and if necessary, sphincterotomy (in a standard manner or with pre-cut technique) and stone removal were carried out. LC was done under general anesthesia and the three-port method was adopted. When gallbladder inflammation was heavy or gallbladder triangle anatomy was not clear, a fourth port was added. An abdominal drainage tube was placed only when the surgeon deemed it necessary.

### Statistical analysis

Statistical analyses were carried out using the statistical package IBM SPSS Statistics for Windows, Version 26.0 (Armonk, NY). To determine whether the continuous variables were normally distributed, the Shapiro–Wilk test was used. Continuous variables were expressed as mean ± standard deviation or median and quartile, where applicable. Differences between the means of groups were evaluated *via* one-way ANOVA, and median values were compared *via* the Kruskal–Wallis test. Categorical data were compared *via* the chi-square or Fisher's exact test, where appropriate. When *p*-values from the one-way ANOVA, Kruskal–Wallis, or *χ*^2^ test results were statistically significant, a generalized linear model was used to test the true influence of the grouping factor on the results by including the grouping factor and other potential confounding factors. Statistical significance was defined as *p* < 0.05.

## Results

As shown in Figure 1, 476 patients were diagnosed with gallstones and CBD stones. After excluding those not meeting the inclusion criteria, 214 patients were enrolled for further analysis. Demographic and clinical data are shown in Table 1. Group 1 consisted of 52 patients, while groups 2 and 3 consisted of 80 and 82 patients, respectively. Age distribution among the three groups was similar, while gender distribution was significantly different with more females in group 1. The three groups did not differ significantly in comorbidity, blood tests, calculus in the neck of the gallbladder, the thickness of the gallbladder wall, common bile duct, stones in the common bile duct, and amylase after ERCP, except for the proportion of those with biliary pancreatitis.

**Table 1:** Demographic and clinic data of patients.

Variables	Group 1 (*n* = 52)	Group 2 (*n* = 80)	Group 3 (*n* = 82)	F/H/*χ*^2^	P*
Age (years, mean ± SD)	50.69 ± 1.853	55.46 ± 1.569	52.04 ± 1.621	2.1	0.125
Gender (M/F)	19/33	42/38	50/32	7.632	0.022
BMI (kg/m^2^, mean ± SD)	23.75 ± 0.42	23.98 ± 0.46	24.38 ± 0.44	0.504	0.605
Epigastric surgery history (*n*,%)	0 (0)	1 (1.25)	0 (0)	1.992	0.369
Hypertension (*n*,%)	9 (17.3)	23 (28.7)	21 (25.6)	2.265	0.322
Diabetes mellitus (*n*,%)	5 (9.6)	8 (10)	8 (9.8)	0.006	0.997
WBC (×10∧9/L, mean ± SD)	6.62 ± 0.40	7.25 ± 0.40	7.75 ± 0.39	1.785	0.17
Total bilirubin [µmol/L, median (P25,P75)]	34.7 (18.23,201.30)	45.5 (23.9,135)	52.4 (20.45,196.75)	0.521	0.771
Direct bilirubin [µmol/L, median (P25,P75)]	24.5 (12.65,109.93)	25.5 (14.3,79.4)	31.7 (9.85,118)	0.2	0.905
ALT (units/L)	119.5 (62.25,285.5)	157 (78,326)	137 (85.5,224.25)	0.686	0.709
AST (units/L)	174.5 (28.5,346)	168 (32,371)	148 (59,377)	0.207	0.902
ALP (units/L)	40.6 (20.025,252.25)	61.3 (21.3,360)	83.3 (20.675,214.75)	1.066	0.587
γ-GT (units/L)	39.9 (7.6,119.75)	82 (6.2,139)	78.85 (9,150.3)	1.945	0.378
Biliary pancreatitis (*n*,%)	0 (0)	12 (15)	6 (7.3)	9.411	0.009
Calculus in the neck of the gallbladder (*n*,%)	7 (13.5)	20 (25)	23 (28)	3.972	0.137
The thickness of gallbladder wall ≥4 mm (*n*,%)	17 (32.7)	26 (32.5)	29 (35.4)	0.177	0.915
Choledocholithiasis (solitary/multiple)	41/11	51/29	57/25	3.397	0.183
Diameter of Choledocholithiasis [mm, median (P25,P75)]	4 (3,6)	4 (2,6)	5 (3,6)	1.964	0.375
Diameter of common bile duct [mm, median (P25,P75)]	9 (8,12)	10 (8,11)	10 (8,12)	1.988	0.37
2 times of ERCP (*n*,%)	0 (0)	1 (1.25)	4 (4.88)	4.731	0.094
Amylase after ERCP (units/L)	250 (126.25,561)	160 (88,396)	214.5 (109,584)	5.523	0.063
4 port technique (*n*,%)	2 (3.8)	3 (3.8)	8 (9.8)	3.05	0.218
The drainage tube (*n*,%)	12 (23.1)	13 (16.3)	25 (30.5)	4.588	0.101
Operative time [min, median (P25,P75)]	65 (55.25,85)	66 (50,80)	80 (60,105)	9.87	0.007
Post-cholecystectomy hospital stay [days, median (P25,P75)]	4 (4,7)	4 (3,6)	4 (3,5)	7.753	0.021
Post-ERCP hospital stay [days, median (P25, P75)]	5 (4.25,8)	6 (5,8)	8 (7,10.25)	52.795	<0.001
Conversion to open cholecystectomy (*n*,%)	0 (0)	2 (2.50)	1 (1.22)	2.053	0.358
Additional ERCP after LC (*n*,%)	1 (1.92)	3 (3.75)	3 (3.66)	0.396	0.821
Major morbidity (*n*,%)	1 (1.92)	0 (0)	1 (1.22)	1.375	0.503
Biliary injury (*n*,%)	0 (0)	0 (0)	0 (0)		
Bile leakage (*n*,%)	1 (1.92)	0 (0)	1 (1.22)	1.375	0.503
Hemorrhage (*n*,%)	0 (0)	0 (0)	0 (0)		

Group 1, LC on the next day after ERCP; Group 2, LC on day 2 and 3 after ERCP; Group 3, LC on day 4 or later after ERCP.

SD, standard deviation; BMI, body mass index; ALT, alanine transaminase; AST, aspartate aminotransferase; ALP, alkaline phosphatase; γ-GT, gamma-glutamyl transpeptidase; LC, laparoscopic cholecystectomy; ERCP, endoscopic retrograde cholangiopancreatography.

*The *p*-values are the results of the comparison between the three groups.

Table 1 shows the operative times among the three groups. This was 65 min [interquartile range (IQR), 55.25–85 min], 66 min (IQR, 50–80 min), and 80 min (IQR, 60–105 min) in groups 1, 2, and 3, respectively and this difference was statistically significant (*p* = 0.007).

Elevated WBC was defined as a WBC count higher larger than 9.5 × 10^9^/L based on the standards for testing instruments in our hospital.

WBC, white blood cell; TB, total bilirubin; DB, direct bilirubin; ALT, alanine transaminase; AST, aspartate aminotransferase; ALP, alkaline phosphatase; γ-GT, gamma-glutamyl transpeptidase; CBD, common bile duct; LC, laparoscopic cholecystectomy; ERCP, endoscopic retrograde cholangiopancreatography.

Univariate analysis (Table 2) showed that male gender, biliary pancreatitis, calculus in the neck of the gallbladder, the thickness of the gallbladder wall ≥4 mm, age, total bilirubin, direct bilirubin, alanine aminotransferase, aspartate aminotransferase, gamma-glutamyl transpeptidase, and different interval time between ERCP and LC were all risk factors.

**Table 2:** Univariate analysis for post-ERCP, postoperative hospital stay, and operation time.

Factors	Post-ERCP hopital stay		Postoperative hospital stay		Operation time	
	z	*p*	z	*p*	z	*p*
Gender						
Female	Reference					
Male	−0.727	0.468	−0.420	0.674	−2.229	0.026
History of surgery						
No	Reference					
Yes	−0.353	0.724	−0.041	0.967	−0.864	0.388
Obesity						
No	Reference					
Yes	−1.764	0.078	−0.204	0.838	−1.858	0.063
Hyperamylasemia						
No	Reference					
Yes	−1.194	0.233	−1.694	0.090	−1.138	0.255
Hypertension						
No	Reference					
Yes	−1.959	0.050	−0.785	0.433	−0.070	0.944
Diabetes mellitus						
No	Reference					
Yes	−0.079	0.937	−0.168	0.866	−1.473	0.141
Coronary heart disease						
No	Reference					
Yes	−0.989	0.322	−1.257	0.209	−0.706	0.480
Biliary pancreatitis						
No	Reference					
Yes	−2.586	0.010	−2.056	0.040	−2.601	0.009
Calculus in the neck of the gallbladder						
No	Reference					
Yes	−0.536	0.592	−0.813	0.416	−3.874	0.000
The thickness of the gallbladder wall ≥4 mm						
No	Reference					
Yes	−1.647	0.099	−1.885	0.059	−2.660	0.008
Number of choledocholithiasis						
Solitary	Reference					
Multiple	−0.556	0.578	−0.321	0.748	−0.446	0.655
The number of ERCP						
1	Reference					
2	−2.775	0.006	−1.099	0.272	−0.633	0.526
Additional ERCP after LC						
No	Reference					
Yes	6.130	0.013	−2.354	0.019		
Elevated WBC						
No	Reference					
Yes	9.788	0.002	−2.838	0.005	−2.610	0.009
Age	−0.048	0.484	0.034	0.616	0.162	0.017
TB	−0.064	0.354	0.274	<0.001	0.243	<0.001
DB	−0.090	0.190	0.307	<0.001	0.257	<0.001
ALT	−0.001	0.985	0.143	<0.001	0.223	0.001
AST	0.001	0.990	0.279	<0.001	0.268	<0.001
GGT	0.096	0.163	−0.065	0.348	0.156	0.022
ALP	0.148	0.031	−0.125	0.068	0.101	0.143
Diameter of CBD stone	0.045	0.512	−0.007	0.916	0.020	0.77
Diameter of CBD	−0.029	0.678	0.069	0.313	0.070	0.306
Group	52.759	<0.001	7.753	0.021	9.87	0.007

A generalized linear model was constructed as shown in Table 3. Biliary pancreatitis [odds ratio (OR) 5.501, *p* = 0.019], calculus in the neck of the gallbladder (OR 10.187, *p* = 0.001), the thickness of the gallbladder wall (OR 5.123, *p* = 0.024), and the interval time between LC and ERCP were risk factors for prolonged operation time. Among these groups, operation time in group 1 and group 2 was similar (OR 0.144, *p* = 0.704), while operation time was significantly longer in group 3 than in group 1 (OR 4.005, *p* = 0.045). The numbers of the conversions to open cholecystectomy were 0, 2, and 1 in groups 1, 2, and 3, respectively, and this difference was not statistically significant. The number of difficult surgeries that needed a four-port technique LC was 13 (6.1%) in total. A drainage tube was required in a total of 50 patients (23.4%). There was no significant difference among the three groups in terms of requiring a fourth port or a drainage tube.

**Table 3:** Factors for prolonged operating time.

Parameter estimation						
Variables	B	Standard error	95% CI		Wald	*p*
Biliary pancreatitis	−20.458	8.7224	−37.554	−3.363	5.501	0.019
	Reference					
Calculus in the neck of the gallbladder						
Yes	16.017	5.0183	6.182	25.853	10.187	0.001
No	Reference					
The thickness of the gallbladder wall ≥4 mm						
Yes	10.136	4.4785	1.359	18.914	5.123	0.024
No	Reference					
Group						
3	10.527	5.2603	0.217	20.837	4.005	0.045
2	−2.043	5.3848	−12.597	8.511	0.144	0.704
1	Reference					

Group 1, LC on the next day after ERCP; Group 2, LC on day 2 and 3 after ERCP; Group 3, LC on day 4 or later after ERCP.

CI, confidence interval.

Post-cholecystectomy complications: The overall morbidity rate was 4.2%, including retention of CBDS (3.3%) and bile leakage (0.9%). The retained stones were successfully removed by additional ERCP after LC and the bile leakage was managed by ultrasound-guided peritoneal puncture and catheter drainage plus antibiotics.

Post-cholecystectomy hospitalization was found to be significantly different among these groups by the Kruskal–Wallis test: 4 days (IQR, 4–7 days), 4 days (IQR, 3–6 days), and 4 days (IQR, 3–5 days), respectively, in groups 1, 2 and 3 (*p* = 0.021). However, as shown in Table 4, after controlling for confounding factors, the generalized linear model showed that only total bilirubin (OR 5.639, *p* = 0.018), elevated amylase after ERCP (OR 4.558, *p* = 0.033), and additional ERCP after LC (OR 8.842, *p* = 0.003) for retained common bile duct stone were risk factors for a prolonged post-cholecystectomy hospital stay.

**Table 4:** Factors for prolonged post-cholecystectomy hospitalization.

Parameter estimation						
Variables	B	Standard error	95% CI		Wald	*p*
Total bilirubin	0.006	0.0023	0.001	0.010	5.639	0.018
Elevated amylase after ERCP						
Yes	0.962	0.4504	0.079	1.845	4.558	0.033
No	Reference					
Additional ERCP after LC						
Yes	4.544	1.5280	1.549	7.539	8.842	0.003
NO	Reference					
Group						
3	−0.623	0.5459	−1.693	0.447	1.300	0.254
2	−0.539	0.5574	−1.631	0.554	0.935	0.334
1	Reference					

Group 1, LC on the next day after ERCP; Group 2, LC on day 2 and 3 after ERCP; Group 3, LC on day 4 or later after ERCP.

ERCP, endoscopic retrograde cholangiopancreatography; LC, laparoscopic cholecystectomy; CI, confidence interval.

Post-ERCP hospitalization was also significantly different among the three groups by the Kruskal–Wallis test: 5 days (IQR, 4.25–8 days), 6 days (IQR, 5–8 days), and 8 days (IQR, 7–10.25 days), in groups 1, 2 and 3, respectively (*p* < 0.001). However, in the generalized linear model, as shown in Table 5, only additional ERCP after LC and interval time were risk factors for prolonged post-ERCP hospitalization. Compared with group 1, group 2 did not have significantly prolonged post-ERCP hospitalization (*p* = 0.384) while group 3 did (*p* < 0.001).

**Table 5:** Factors for prolonged post-ERCP hospitalization.

Parameter estimation						
Variables	B	Standard error	95% CI		Wald	*p*
Additional ERCP after LC						
Yes	4.613	1.8563	0.975	8.251	6.175	0.013
No	Reference					
Group						
3	3.449	0.6747	2.127	4.772	26.136	<0.001
2	0.602	0.6915	−0.753	1.958	0.759	0.384
1	Reference					

Group 1, LC on the next day after ERCP; Group 2, LC on day 2 and 3 after ERCP; Group 3, LC on day 4 or later after ERCP.

ERCP, endoscopic retrograde cholangiopancreatography; LC, laparoscopic cholecystectomy; CI, confidence interval.

Additionally, we collected articles about the optimal timing for LC after ERCP shown in Table 6. The conversion rate to open surgery varies from 0% to 25% in various studies, and no significant difference was observed among different intervals. The complication rate varies from 0% to 25%, and two studies showed that performing LC within three days reduced the complication rate. Similarly, several studies reported that operative times were reduced when doctors perform LC within three days after ERCP was done. What is more, shorter hospitalization was observed when LC was done after ERCP was finished within one day. Interestingly, we did not find any article that reported any advantage when we prolong the interval between LC and ERCP on the aspect of conversion rate, complication, operative time, and hospitalization.

**Table 6:** 

Demographics			Outcomes									
Study	Sample	Group: time intervals between ERCP and LC	Complications *n* (%)	*P*	Mortality *n* (%)	*p*	Conversion *n* (%)	*p*	Operating time min	*p*	Length of stay day	*p*
de Vries (17)	23	<2 weeks	4 (17.4)	0.66	0	NA	1 (4)	0.052	75 (40–150)	0.35	NA	NA
	13	2–6 weeks	1 (6.7)		0		5 (31)		75 (40–200)			
	45	>6 weeks	5 (11.1)		0		7 (16)		65 (30–135)			
Bostanci (16)	95	<2 days	5 (1)	NA	0	NA	13 (13.7)	0.472	52.5 ± 28.9	0.511	2.2 ± 1.8	0.641
	100	3–42 days	11 (3)		0		11 (11.0)		58.4 ± 33.6		2.6 ± 3.6	
	113	>42 days	9 (2)		0		19 (16.8)		53.52 ± 30.0		2.7 ± 6.1	
Borreca (15)	36	<24 h	0 (0)		0	NA	0 (0)	0.33	76	0.7	4.7	0.001
	29	24–72 h	2 (7)		0		1 (3.5)		80		8	
	28	>72 h	2 (7)		0		0 (0)		84		10.7	
Kwon (14)	148	<2 weeks	21 (14.2)	0.71								
	71	2–6 weeks	13 (18.4)									
	38	>6 weeks	6 (15.6)									
Wild (13)	65	same day	NA	NA	0 (0)	0.958	8 (12)	0.862	88 (70–119)	0.457	3 (2–7)	<0.001
	175	>1 day	NA	NA	2 (1.1)		24 (14)		89 (68–125)		5 (3–7)	
El Nakeeb (12)	55	<3 days	6 (10.9)	NA	NA	NA	5 (9.1)	0.75	50 (30–180)	0.2	1 (15)	0.06
	55	>1 month	9 (16.4)				6 (10.9)		60 (30–180)		1 (1–6)	
Aziret (11)	30	48–72 h	3 (10.0)	0.012			1 (3.3)	0.15	60 (60–72.5)	0.003	4 (3–7)	0.332
	25	72 h - 6 weeks	11 (44.0)				4 (16.0)		85 (65–110)		3 (2–9.5)	
	30	6–8 weeks	11 (36.7)				5 (16.7)		85 (60–101.2)		3 (2–6)	
Trejo-Avila (10)	40	<24 h	2 (5)	NA	0	NA	0 (0)	NA	98.7 ± 38.95	0.078	2 (2–3)	<0.001
	47	>24 h	4 (8.5)		0		0 (0)		85.9 ± 27.31		4 (4–7)	
Senocak (9)	25	<2 weeks	15 (60)	0.289	0	NA	5 (20.0)	0.646	80 (60–120)	0.861	3 (1–6)	0.634
	20	2–6 weeks	12 (60)		0		5 (25.0)		71 (60–101)		2.5 (1–5)	
	22	>6 weeks	13 (59.1)		0		3 (13.6)		75 (68–96)		4 (2–5)	
Abdalkoddus (8)	62	<2 weeks	0 (0)	0.7	0	NA	4 (6.8)	0.7	75 (55–95)	0.4	2 (1–6)	0.08
	90	2–6 weeks	2 (2.2)		0		3 (3.6)		80 (55–103)		2 (1–5)	
	292	>6 weeks	7 (2.4)		0		16 (6)		70 (50–93)		1 (0–5)	
Gao (7)	52	1–3 days	1 (1.92)	0.013	0	NA	1 (1.92)	0.298	50.03 ± 10.65	<0.001	7.18 ± 1.23	0.776
	51	4–7 days	8 (15.69)		0		3 (5.88)		69.79 ± 15.31		7.25 ± 1.26	

ERCP, endoscopic retrograde cholangiopancreatography; LC, laparoscopic cholecystectomy; NA, not available.

## Discussion

Cholecystolithiasis complicated with choledocholithiasis is a common clinical situation. Several guidelines recommend that patients diagnosed with CBDSs be offered stone extraction, if possible (3, 18). LCBDE and LC plus intra-operative ERCP are both excellent strategies. However, they are not easily accessible in all centers. Thus, pre-operative ERCP plus LC is a commonly used approach. However, pre-operative ERCP can make cholecystectomy difficult due to bacterial colonization in the bile ducts resulting in infection of the hepatoduodenal ligament (19), and hence high-grade adhesions in the gallbladder triangle (16). Many clinicians have explored the ideal interval time from ERCP to LC, as shown in Table 6, but this is still controversial to date. Based on data shown in Table 6, this approach is safe because only two deaths have been reported so far and the complication rate is not high. The conversion rate to open surgery varies from 0% to 25% in various studies. Some studies have suggested that performing LC within three days post-ERCP is a better option because of fewer complications, shorter operative time, and shorter hospitalization. Aziret et al*.,* and Gao et al*.* reported that performing LC within one to three days of ERCP was associated with a lower complication rate as compared with LC done four days or later. They also found that performing LC within one to three days of ERCP shortened the operative time (7, 11). Borreca et al*.*, Wild et al*.*, and Trejo-Avila et al*.* suggested performing LC within 24 h post-ERCP so that hospitalization could be significantly shortened (10, 13, 15).

LC is a gold standard operation for gallstones with or without acute cholecystitis, even with severe inflammation. We enrolled patients with acute cholecystitis, acute cholangitis, and mild biliary pancreatitis and had satisfactory outcomes. Our study did not find any difference in the conversion rate to open surgery or major operative complications among the three groups. The overall conversion rate and LC-related complications were both 1.4%. These were relatively low and consistent with the results of a study by Borreca et al*.* (15). As reported by Aziret et al*.* and Gao et al*.*, operative time was significantly longer in patients receiving LC four days or later after ERCP (7, 11). These results indicate that LC should be done within three days after ERCP. We believe inflammation and adhesion around the gallbladder triangle caused by ERCP and by cholecystitis itself are similar. Early LC (performed within 1–3 days) has been recommended for acute cholecystitis to improve patient outcomes including fewer complications, shorter hospital stay, and hospitalization costs (20).

We found in our study that patients with biliary pancreatitis, calculus in the neck of the gallbladder, and gallbladder wall thickness ≥4 mm before ERCP had longer operative times, indicating that ERCP is not the only factor responsible for prolonged operative time. Extensive inflammation caused by gallstones, CBDSs, and bacteria in the bile can also make LC very difficult (17). Surgeons operating on patients with gallstones and co-existing CBDSs should be mindful of the fact that factors other than ERCP may contribute to difficulties during surgery, and should consider switching to open surgery if needed to reduce postoperative complications or delay surgery till inflammation subsides.

Length of hospital stay is an important standard because longer hospital stays mean higher hospitalization costs (15). Post-cholecystectomy hospital stay was similar among three groups, while post-ERCP hospital stay was significantly longer in group 3. These results indicated that early LC does not prolong post-cholecystectomy hospital stay. The time taken for waiting for LC after ERCP significantly prolongs the length of hospital stay after ERCP, and such a wait is probably unnecessary.

Based on our study and studies published previously, performing LC in the same admission as ERCP is safe and effective, even though these patients may have underlying acute cholecystitis, acute cholangitis, or mild biliary pancreatitis. Further, recurrent biliary symptoms and biliary complications including gallbladder perforation and recurrent choledocholithiasis can be reduced (12). Performing LC early within three days rather than four days or later after ERCP is recommended because of the reduced operating time and hospital stay, without increased complications and conversion rates. In patients with risk factors for difficult surgery, especially those with a high possibility of conversion to open cholecystectomy, attention is needed during surgery to reduce biliary injury. Percutaneous transhepatic gallbladder puncture drainage may have to be considered in some patients to overcome severe acute inflammation, prior to a second surgery (21).

The limitation of our study is that it was a single center retrospective study. The limitations of retrospective analysis are inevitable. A large, multicenter, randomized controlled trial is needed to address whether LC done within three days of ERCP is superior in terms of fewer post-cholecystectomy complications and shorter hospital stay as compared with LC done four days or later after ERCP.

In conclusion, LC performed within three days after ERCP is safe for patients with gallstone coexisting CBD stones, even in the presence of acute cholecystitis, acute cholangitis, and mild biliary pancreatitis, and is associated with fewer post-cholecystectomy complications and shorter hospital stay.

## Data Availability

The raw data supporting the conclusions of this article will be made available by the authors, without undue reservation.
